# Supplementary material for: Edematous severe acute malnutrition is characterized by hypomethylation of DNA
Source: Nat Commun. 2019 Dec 19;10:5791. doi: 10.1038/s41467-019-13433-6 (PMC6923441; doi:10.1038/s41467-019-13433-6)
Supplement: Supplementary file 2 — Supplementary Information [file 41467_2019_13433_MOESM2_ESM.pdf]

Edematous Severe Acute Malnutrition is Characterized by Hypomethylation of DNA

Schulze, *et. al.*

Supplementary Figures and Tables

SUPPLEMENTARY FIGURES

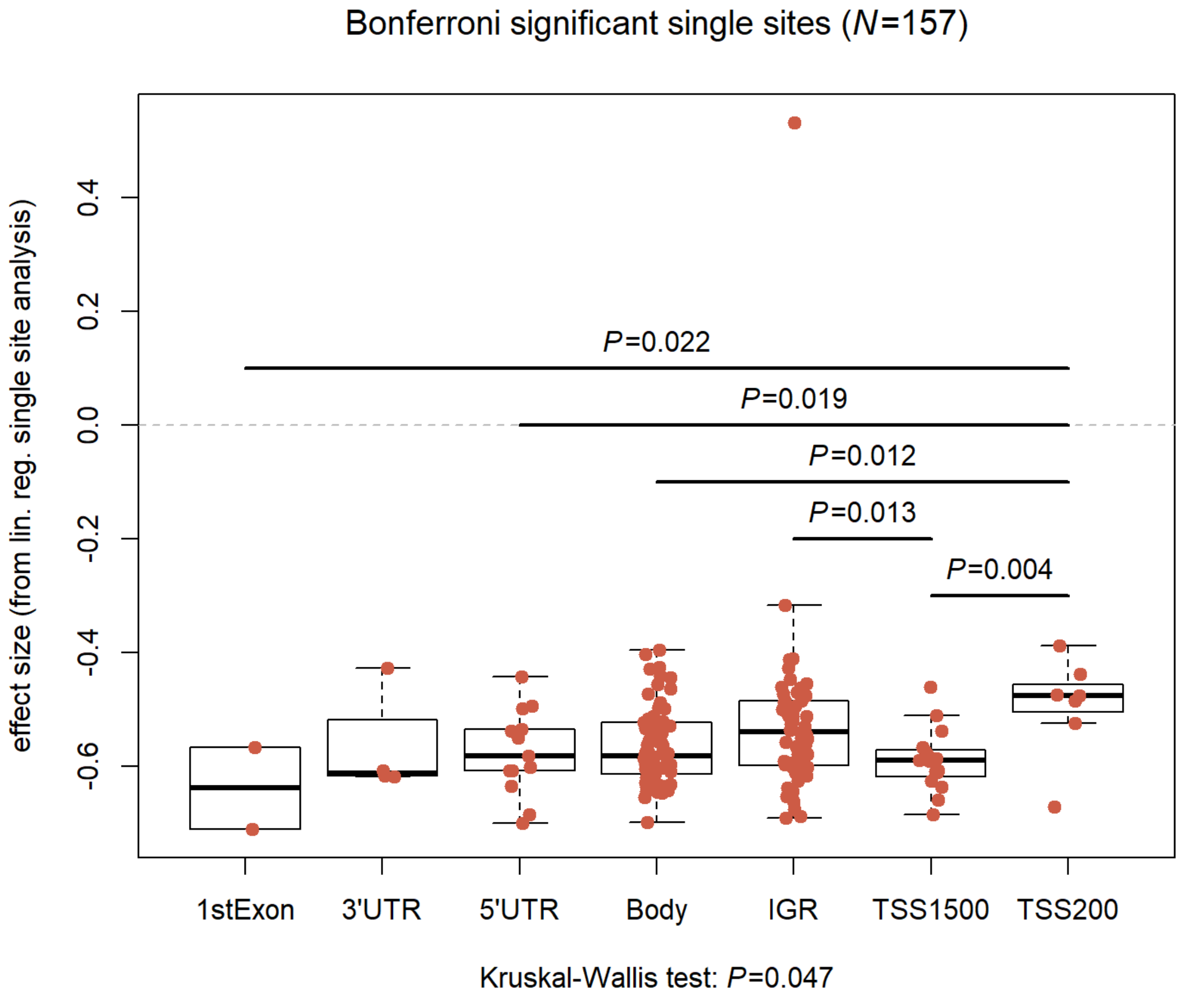

Supplementary Figure 1. Effect size by gene context annotation. Effect sizes were taken from single site linear regression analysis. Dunn's test of multiple comparisons using rank sums was used assess statistical difference between individual categories. Boxplot center lines are medians, box boundaries are first and third quartile, and whiskers extend to data points found within 1.5 times the length of inter quartile range from the median.

3'UTR = 3-prime untranslated region; 5'UTR = 5-prime untranslated region; IGR = intergenic region; TSS1500 = 1,500 bp distance to transcription start site; TSS200 = 200 bp distance to transcription start site.

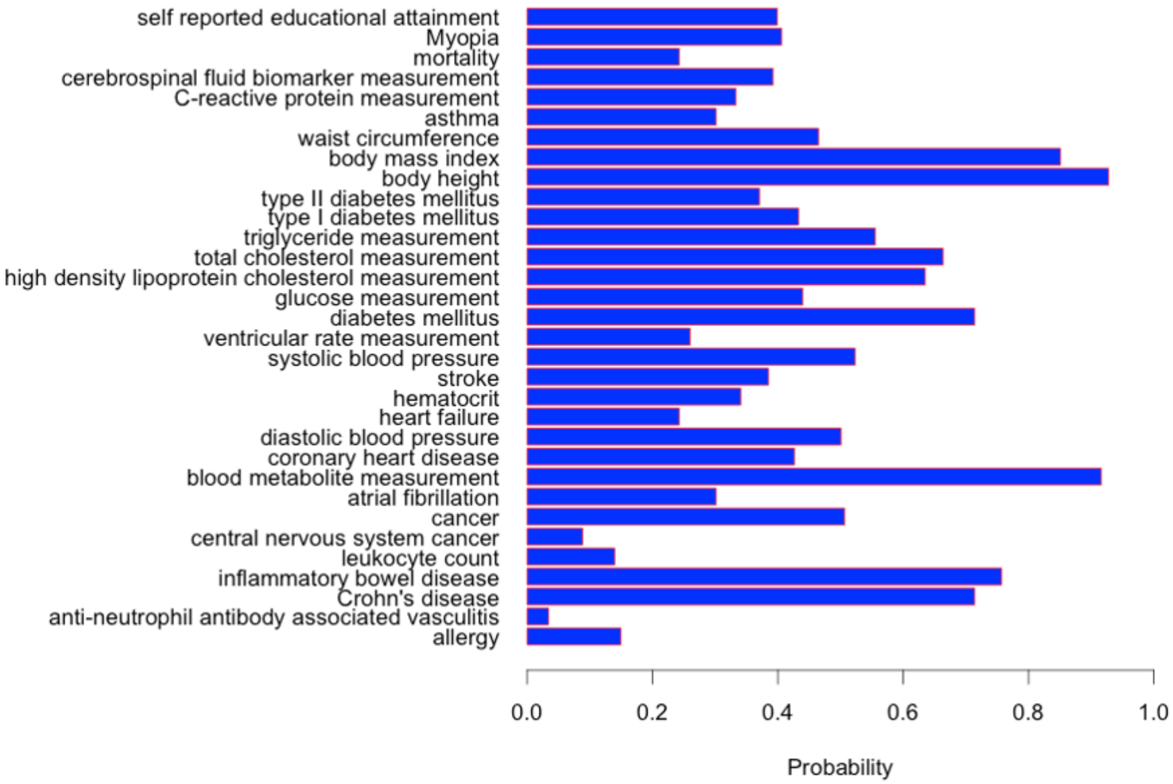

Supplementary Figure 2. Experimental Factor Ontology occurrences in the GWAS catalog.

The horizontal axis shows the hypergeometric probabilities of identifying each of the experimental factor ontologies (EFOs) listed along the vertical axis among a random set of 237 genes selected from the 20,622 genes associated with probes on the Illumina 450K array.

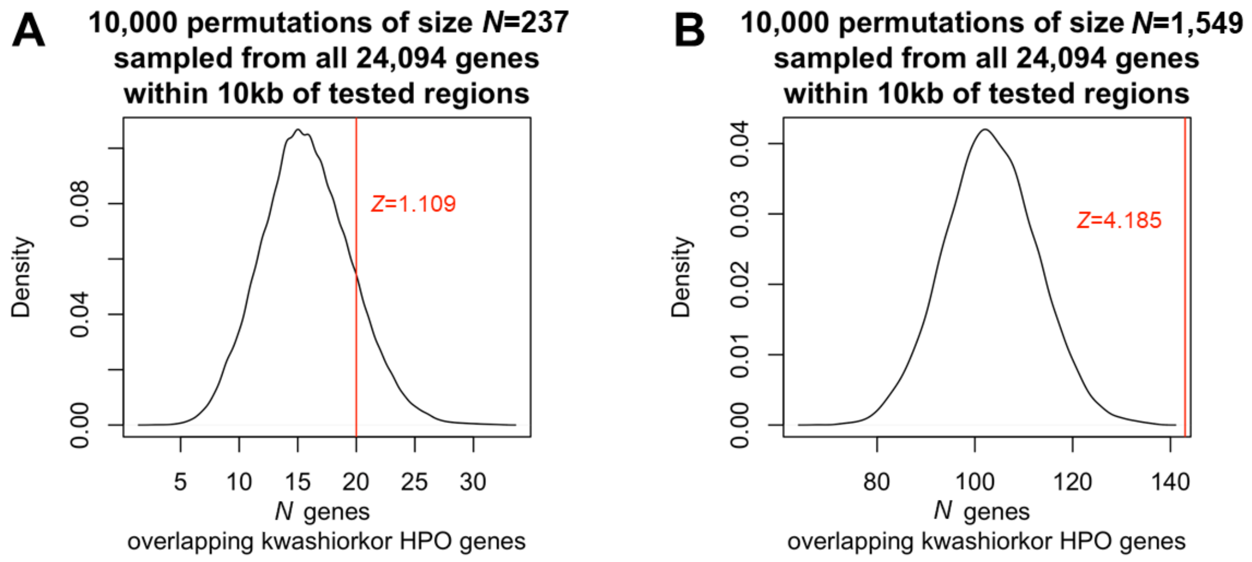

Supplementary Figure 3. Overlap between kwashiorkor HPO and DMC genes. Random samples equal in size to Bonferroni-significant DMC genes (**A**) or those DMC genes with an FDR smaller than 0.01 (**B**) were drawn from the set of genes located within 10 kb of tested regions to estimate the distribution of overlap between these gene sets and genes linked to kwashiorkor HPO terms. Red vertical lines indicate the overlap with non-randomly selected DMC genes found in our study.

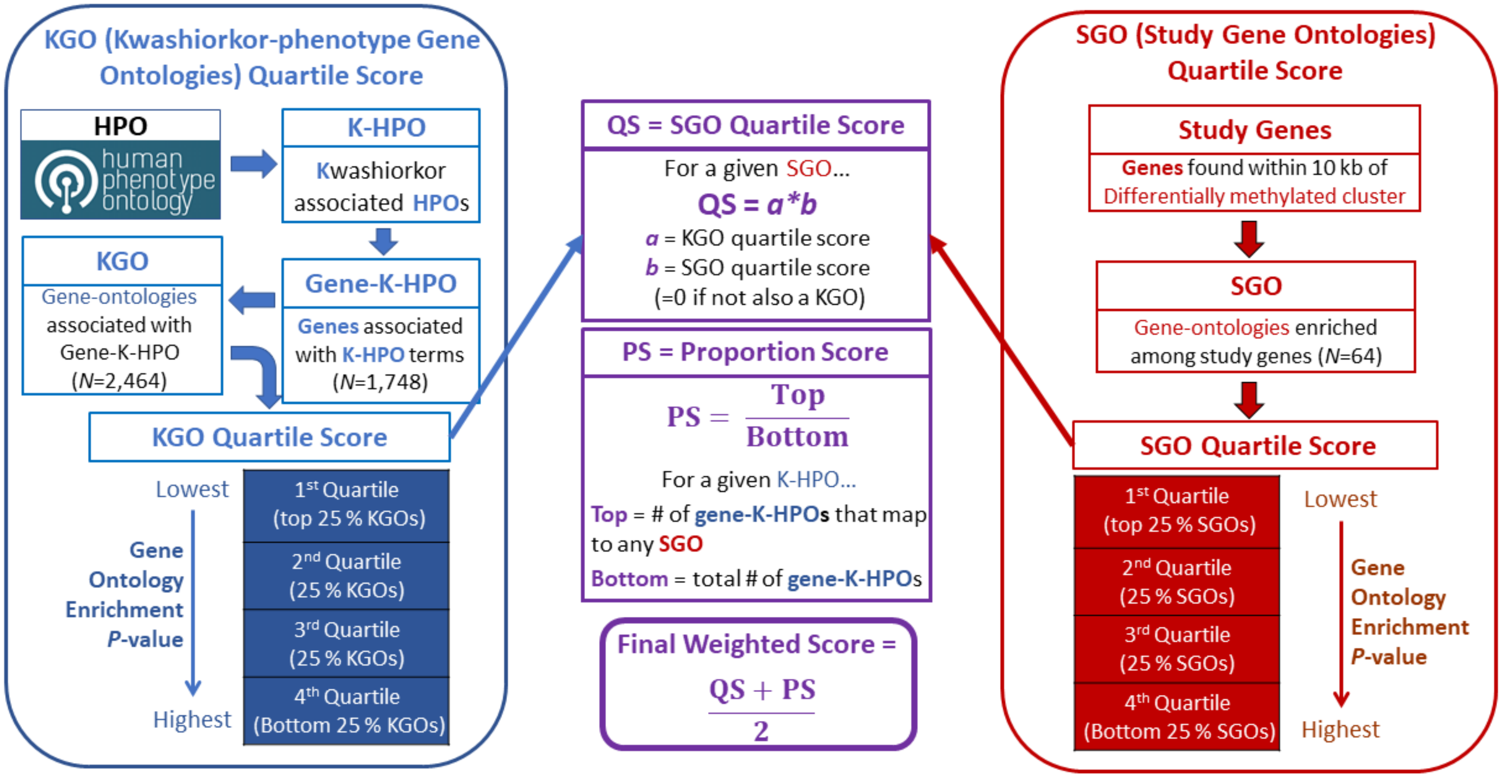

Supplementary Figure 4. Gene Ontology analysis. The flowchart shows the derivation of quartile scores (QS) for each study gene-ontology (SGO), and the proportion score (PS) calculated for each phenotype associated with kwashiorkor (each K-HPO). The final weight score is calculated for each K-HPO-SGO pair.

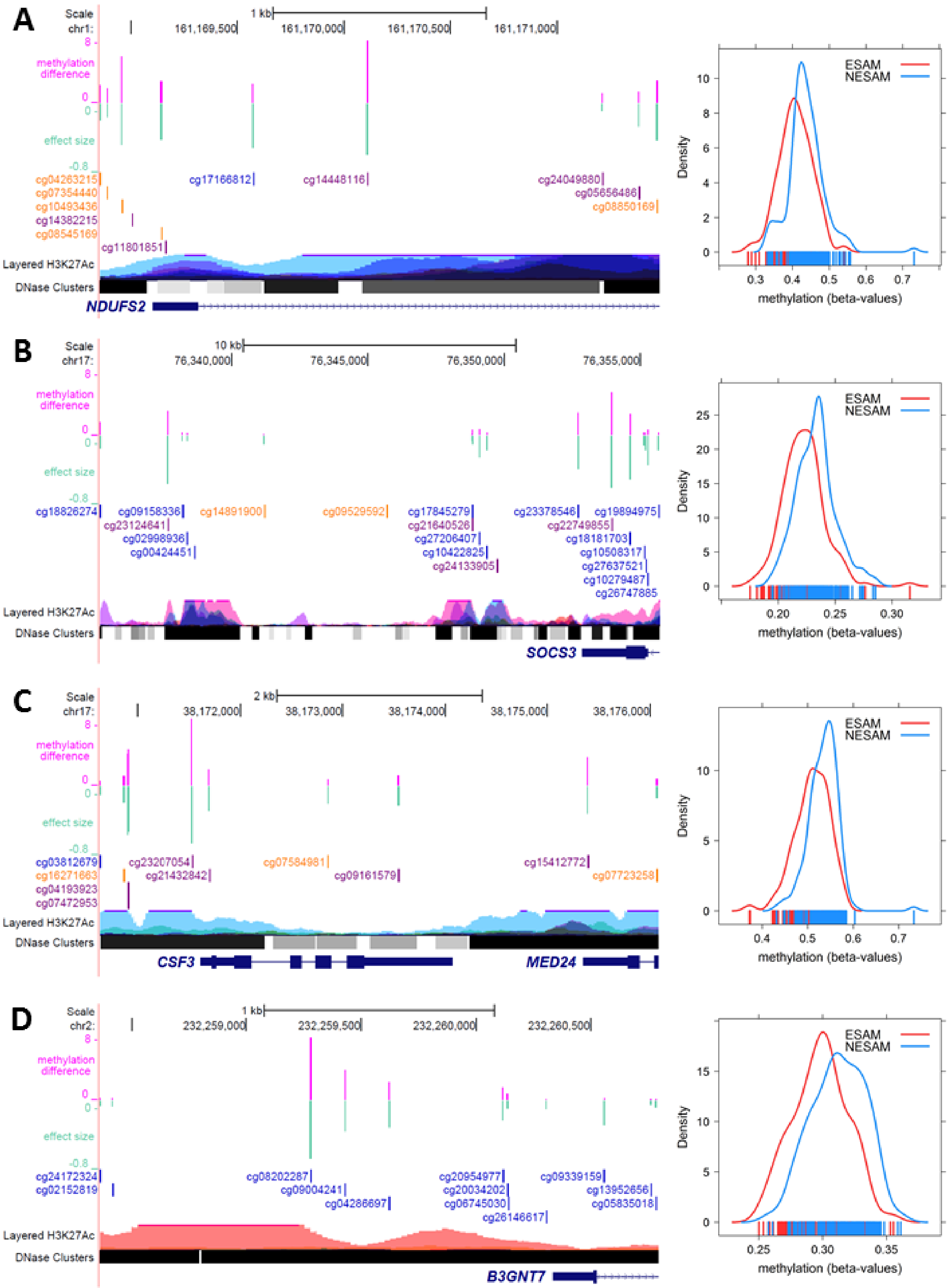
A
B
C
D
Scale
methylation difference
effect size
Layered H3K27Ac
DNase Clusters
NDUFS2
SOCS3
CSF3
MED24
B3GNT7
ESAM
NESAM
Density
methylation (beta-values)

Supplemetnary Figure 5. Differentially methylated clusters (DMCs) from DC analysis. (**A-D**) UCSC Genome Browser tracks (left) of four significant DMCs, accompanied by density plots (right) of the methylation beta-values in these clusters for ESAM and NESAM samples. Methylation difference (magenta) was calculated as: $|\text{mean beta-values}_{\text{ESAM}} - \text{mean beta-values}_{\text{NESAM}}| * 100$. Effect sizes (green) are the coefficients from the linear regression analysis used to determine differential methylation. Probes on the 450K array are indicated by their cg probe IDs. Tracks depicting histone 3 lysine 27 acetylation (H3K27Ac) and DNase I sensitivity (DNase Clusters) were as provided by ENCODE and the UCSC Genome Browser and are both indicators of transcriptionally active genome regions and characterize illustrated DMCs. The Layered H3K27Ac track indicates the level of histone acetylation in multiple tissues shown in different colors. The darker the bar in the DNase Clusters track, the more cell types have been identified to be sensitive to DNase I at the corresponding locus. $N_{\text{ESAM}} = 164$ samples, $N_{\text{NESAM}} = 145$ samples. Source data for density plots are provided as Source Data file.

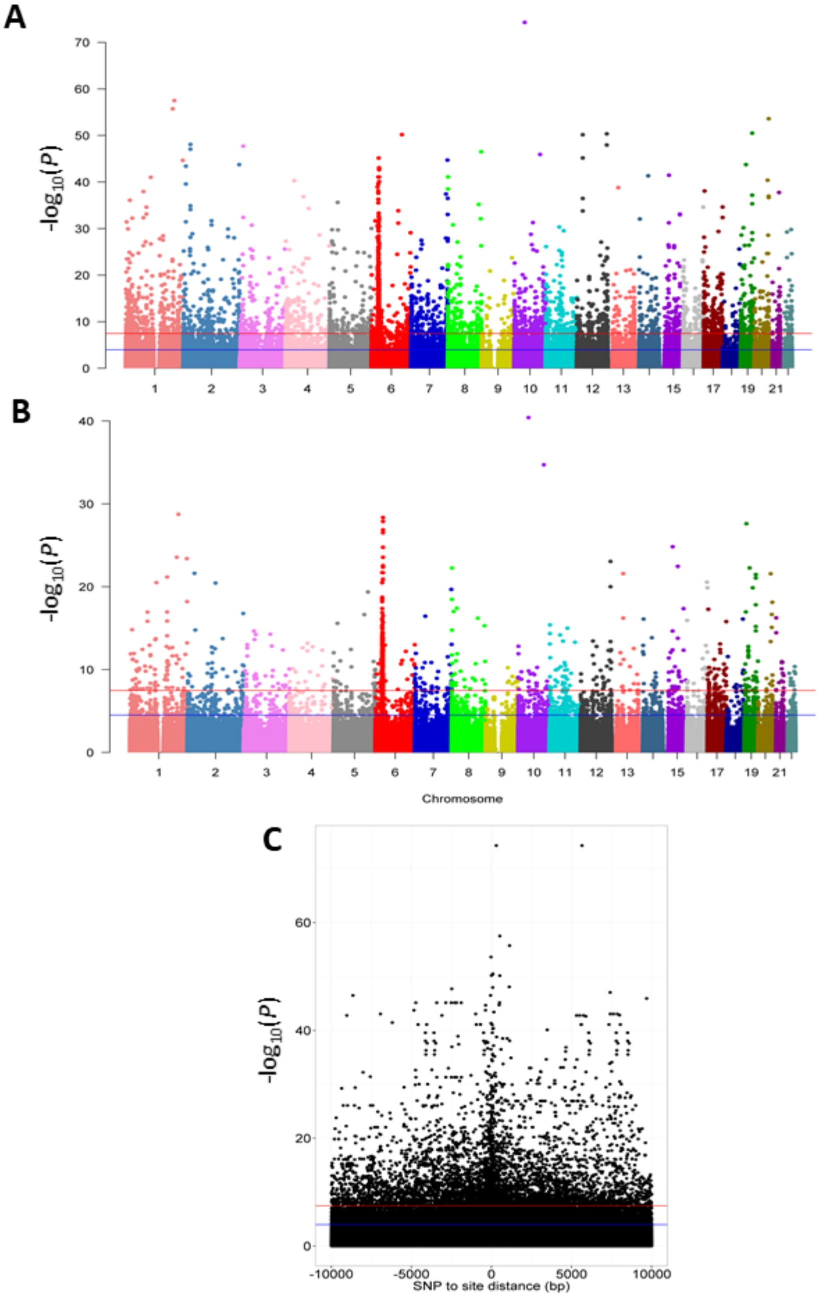

Supplementary Figure 6. Cis-methylation quantitative trait loci (meQTLs) in Jamaican SAM samples. Manhattan plot of cis meQTLs in DC (**A**; *N* = 90) and DL samples (**B**; *N* = 48) are shown. **C -** Scatterplot of –log(*P*) values of cis meQTLs (y-axis) against distance between SNP and CpG probe (x-axis). Red line represents genome-wide significance ($P < 5 \times 10^{-08}$); the blue line represents an FDR < 0.01.

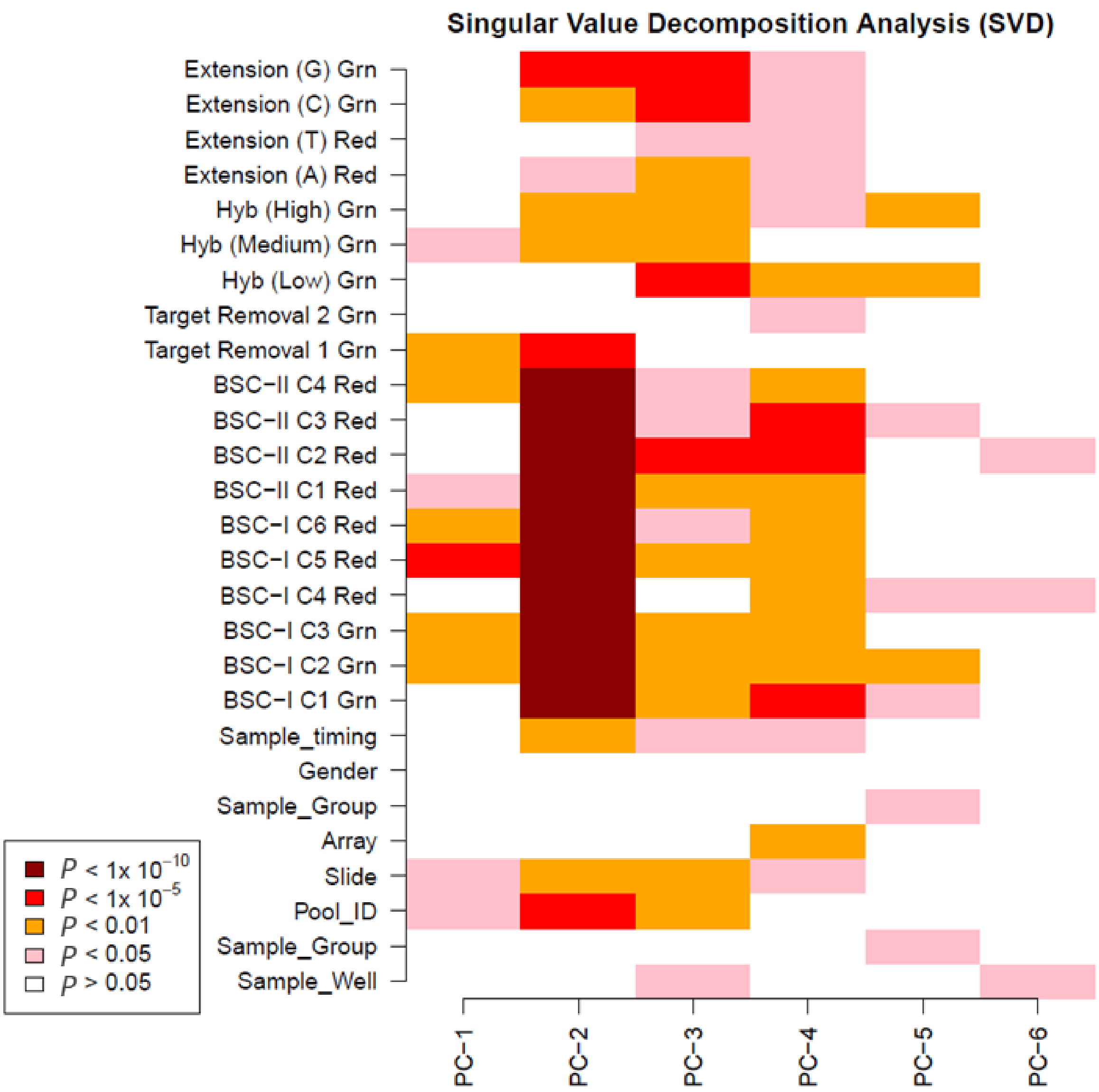

Supplementary Figure 7. Single Value decomposition (SVD) analysis of probe variability (COMBAT).

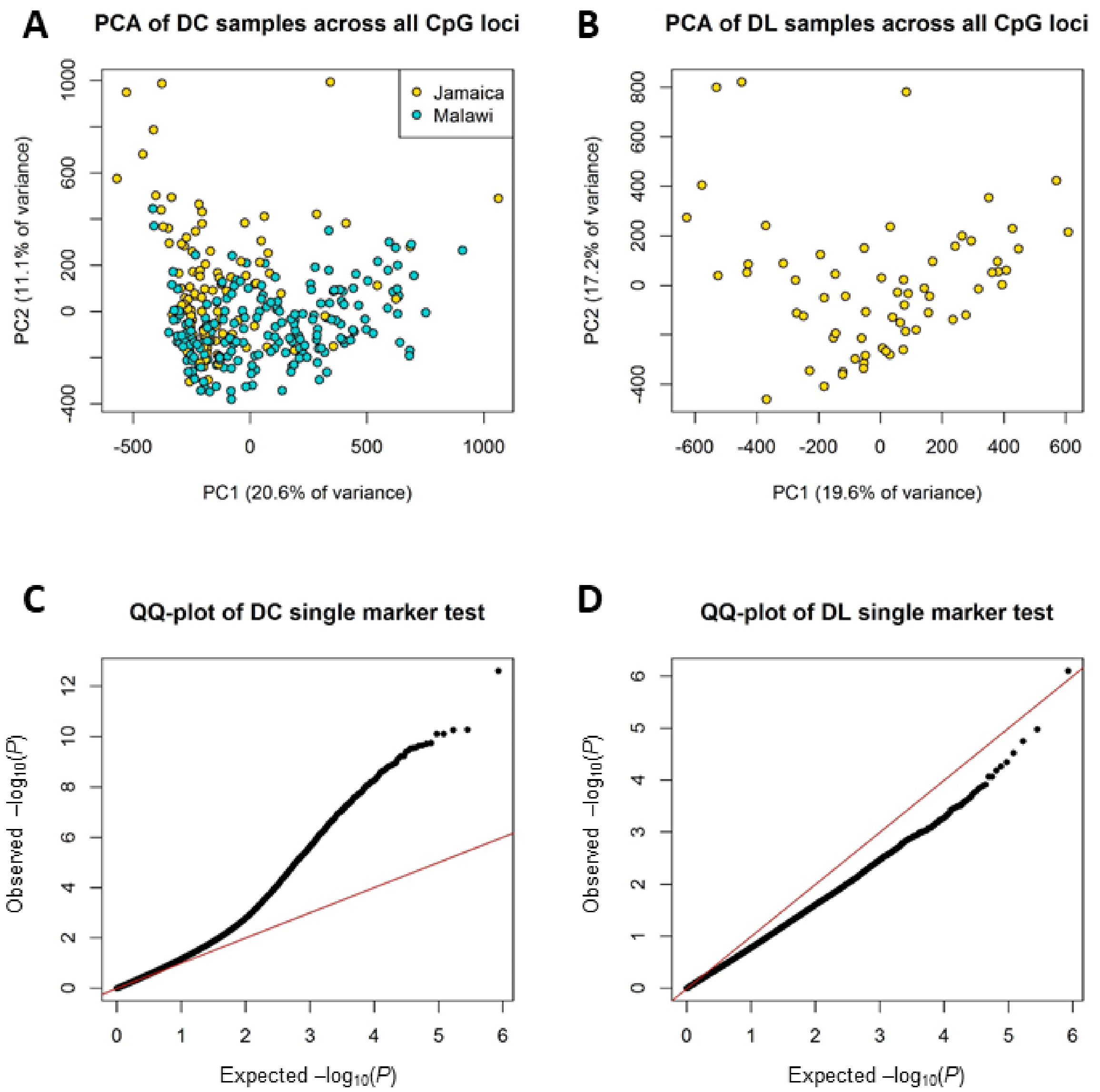

Supplementary Figure 8. PCA and QQ plots from methylation analysis. Results show loci passing filtering thresholds (*N* = 420,500 CpG probes). (**A**) DC samples colored by country of origin; showing the first (PC1) and second (PC2) principal components. (**B**) DL samples. Quantile-Quantile plots (QQ-plots) of nominal *P*-values from single marker analysis of DC samples (**C**) and DL samples (**D**).

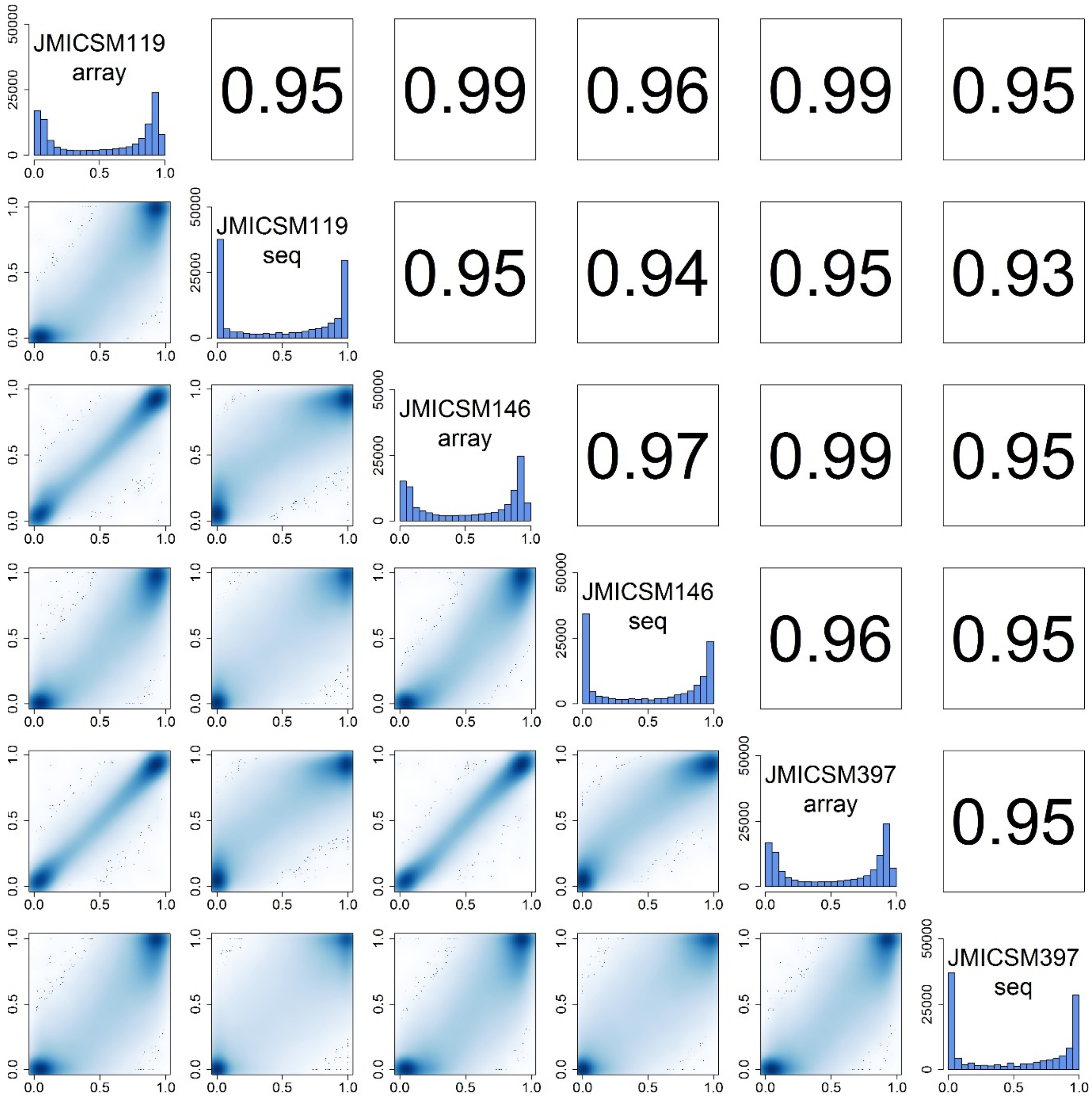

Supplementary Figure 9. Pearson correlation between array and bisulfite sequencing results. Correlations are based on 116,989 CpG sites targeted by both technologies with ≥10X sequencing coverage in all samples. Large numbers in individual boxes indicate the Pearson correlation coefficient between two samples. X- and Y-axes of smoothed scatterplots and X-axes in histograms are methylation proportions, i.e. the number of methylated CpG sites divided by the total number of sequencing reads at a given locus. Histogram Y-axes are frequencies.

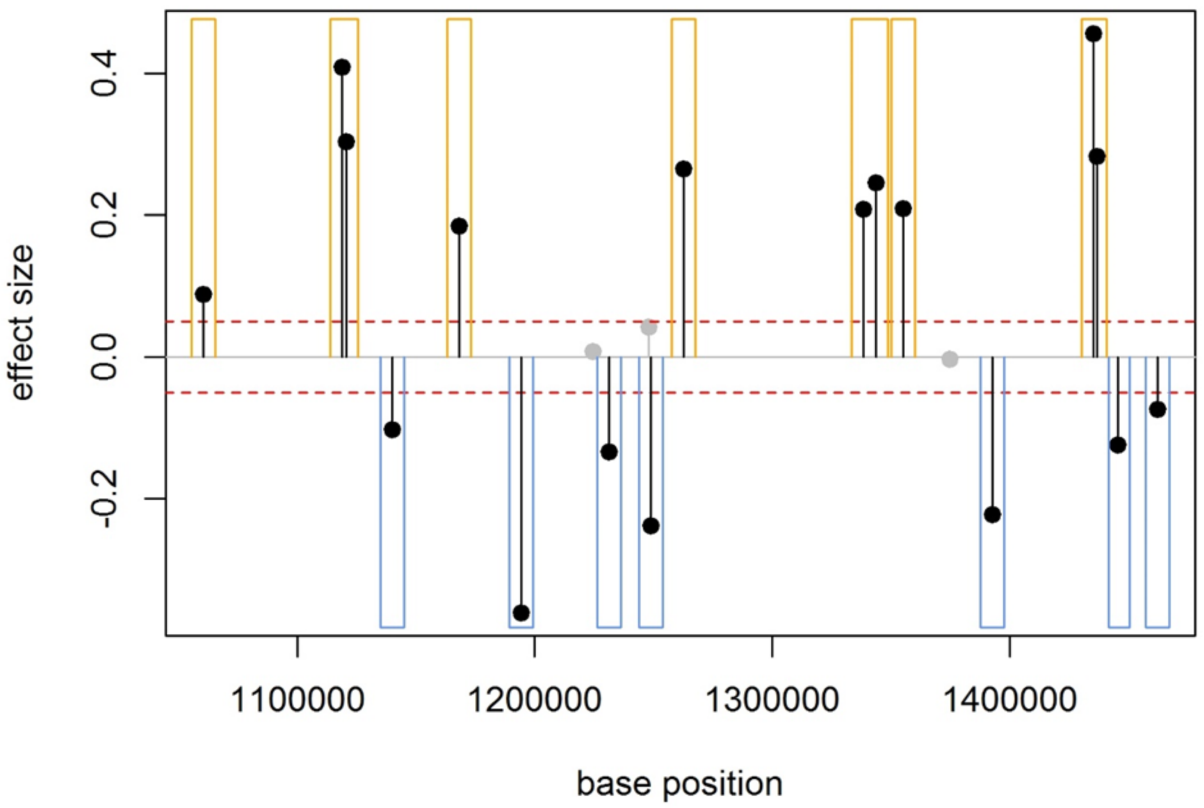

Supplementary Figure 10. Delineation of Differentially Methylated cluster (DMCs). Neighboring probes with the same direction of effect were binned into the same cluster if the distance between them was less than 10 kb. Probes with absolute effect sizes smaller than 0.05 (red dashed line) were excluded (depicted as greyed probes). Hypermethylated clusters are marked in orange, hypomethylated ones in blue.

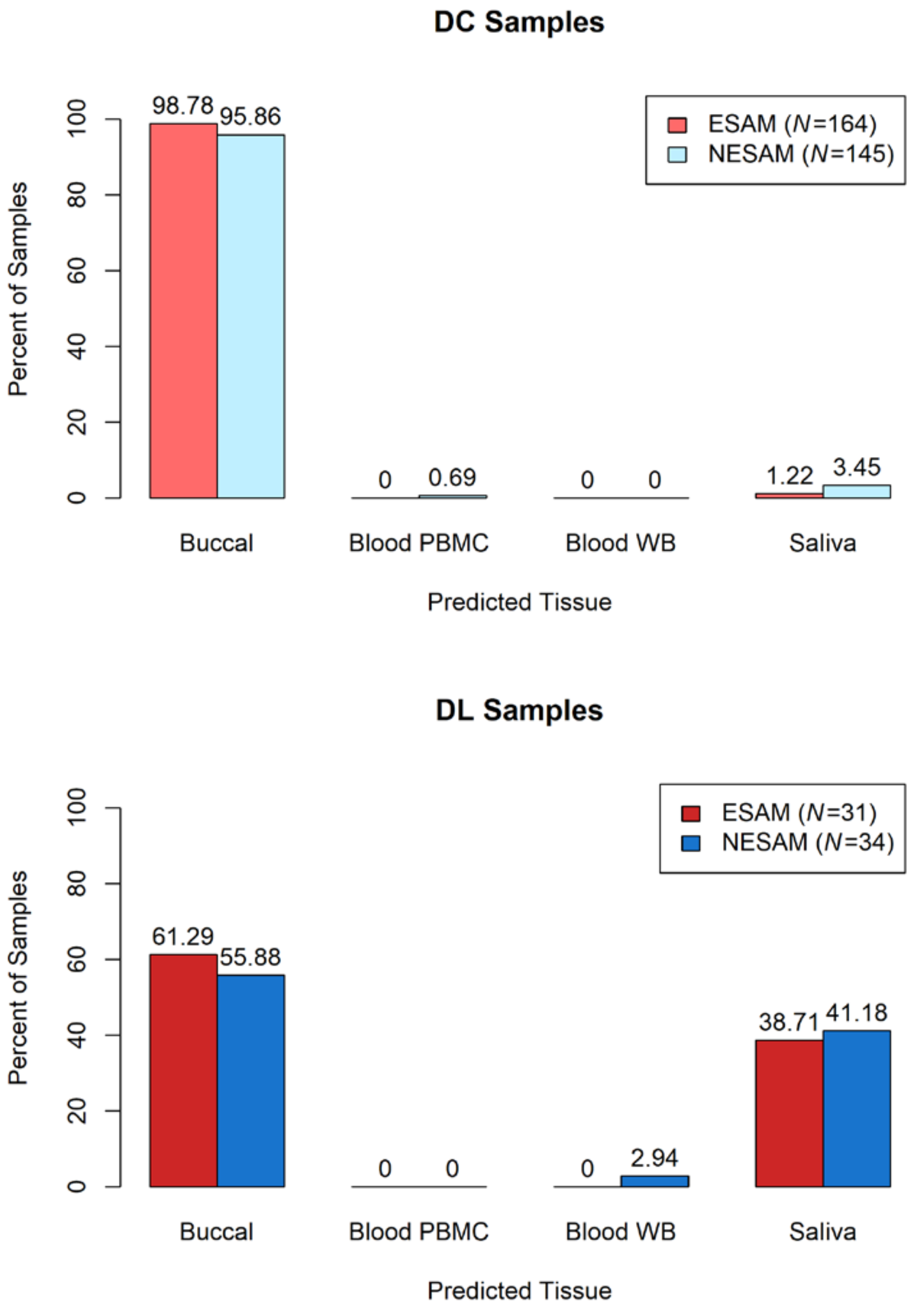

Supplementary Figure 11. Horvath calculator results for tissue composition. Predictions are separated by DC (top) and DL (bottom) samples. PBMC = peripheral blood mononuclear cells; WB = whole blood.

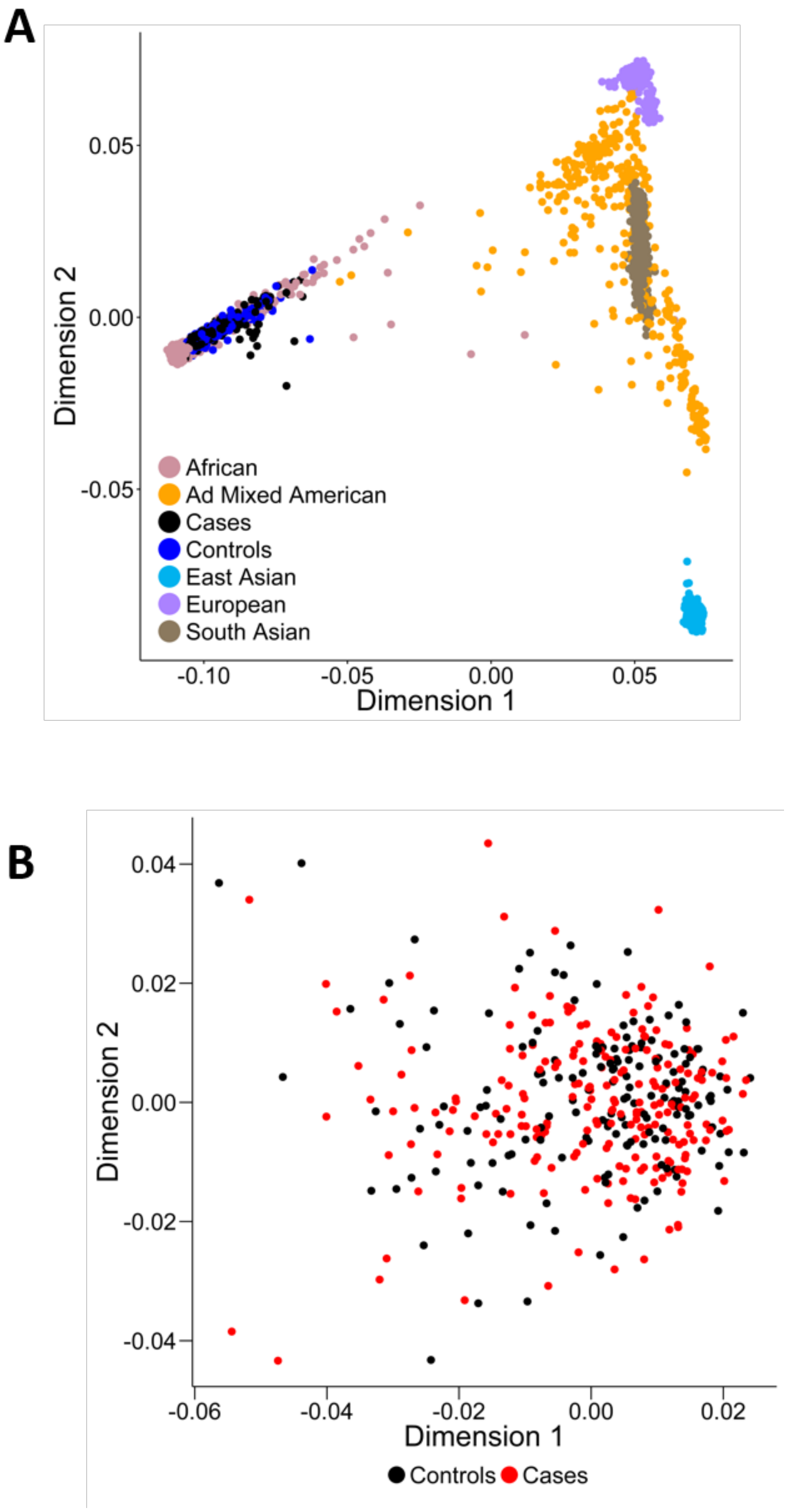

Supplementary Figure 12. Multidimensional scaling plots of genotyped Jamaican samples (*N* = 365). Plots are shown merged with 2504 samples from 1000 Genomes Phase 3 Super Populations (**A**) and by themselves (**B**).

SUPPLEMENTARY TABLES

Sample Demographics by Sample Timing

	DC (*N*=309)			DL (*N*=65)		
	**ESAM (*N*=164)**	**NESAM (*N*=145)**	***P***	**ESAM (*N*=31)**	**NESAM (*N*=34)**	***P***
Males, *N* (%)	83 (50.6)	77 (53.1)	NS[X]	15 (48.4)	20 (58.8)	NS[X]
Mean age (years) ± SE	1.31 ± 0.004	1.2 ± 0.004	NS[T]	30.88 ± 0.283	27.64 ± 0.217	NS[T]
Age range (years)	0.25 – 3.08	0.42 – 3.58	–	17.08 – 49.58	18.33 – 45.00	–
Mean WAZ	-2	-2.77	<0.001[T]	-2.45	-3.92	<0.001[T]
Mean WHZ	-0.89	-1.59	<0.001[T]	-1.64	-2.81	<0.001[T]
Mean MUAC ± SE	12.42 ± 0.013	10.88 ± 0.007	<0.001[T]	–	–	–
Antibiotics at diagnosis, *N* (%)	29 (17.7)	19 (13.1)	NS[X]	–	–	–
HIV+, *N* (%)	3 (1.8)	6 (4.1)	NS[F]	–	–	–
Diarrheal illness, *N* (%)	66 (40.2)	58 (40.0)	NS[X]	–	–	–

DC Samples Demographics by Country

	Jamaica (*N*=109)		Malawi (*N*=200)		***P***
	**ESAM (*N*=61)**	**NESAM (*N*=48)**	**ESAM (*N*=103)**	**NESAM (*N*=97)**	
Males, *N* (%)	31 (50.8)	33 (68.8)	52 (50.5)	44 (45.4)	NS[X]
Kwashiorkor, *N* (%)	49 (80.3)	–	80 (77.7)	–	–
Marasmic-Kwashiorkor, *N* (%)	12 (19.7)	–	23 (22.3)	–	–
Marasmus, *N* (%)	–	37 (77.1)	–	95 (97.9)	–
Undernourished, *N* (%)	–	11 (22.9)	–	2 (2.1)	–
Mean age (years) ± SE	0.83 ± 0.008	1.16 ± 0.013	1.59 ± 0.007	1.22 ± 0.007	<0.001[A]
Age range (years)	0.25 – 2.75	0.42 – 2.83	0.50 – 3.083	0.42 – 3.58	–
Mean WAZ	-1.88	-2.39	-2.07	-2.96	<0.001[A]
Mean WHZ	-0.03	-0.45	-1.4	-2.14	<0.001[A]
Mean MUAC ± SE	–	–	12.42 ± 0.013	10.88 ± 0.007	<0.001[A]
Antibiotics at diagnosis, *N* (%)	–	–	29 (28.2)	19 (19.6)	NS[X]
HIV+, *N* (%)	–	–	3 (2.9)	6 (6.2)	NS[F]
Diarrheal illness, *N* (%)	–	–	66 (64.1)	58 (59.8)	NS[X]

Supplementary Table 1. Sample Demographics: SE = standard error; WAZ = weight for age Z score; WHZ= weight for height Z score; MUAC = mid upper arm circumference; X = Chi-squared test; T = t test; A = analysis of variance; F = Fisher's exact test; NS = not significant. Source data are provided as a Source Data file.

	SAM meQTLs	
	**disease-context-dependent**	**not disease-context-dependent**
**ARIES meQTL in any data subset***	9	200
**not ARIES meQTL in any data subset***	153	1523

Supplementary Table 2. MeQTLs found in ARIES dataset. Fisher's exact $P = 0.018$. *ARIES data subsets: (1) cord blood; peripheral blood: (2) childhood (7 years), (3) adolescence (15-17), (4) pregnancy, (5) middle age.

Covariates	Genome inflation factor (lambda)
Age, Gender, PC1	1.16
Age, Gender, PC1, PC2	1.34
Age, Gender, PC2, PC2, PC3	1.41
Age, Gender, Location	1.24
Age, Gender, Location, PC1	1.48
Age, Gender, Location, PC1, PC2	1.62
Age, Gender, Location, PC1, PC2, PC3	1.70

Supplementary Table 3. Genome-wide inflation in differential methylation analysis.
