# Supplementary material for: Edematous severe acute malnutrition is characterized by hypomethylation of DNA
Source: Nat Commun. 2019 Dec 19;10:5791. doi: 10.1038/s41467-019-13433-6 (PMC6923441; doi:10.1038/s41467-019-13433-6)
Supplement: Supplementary file 3 — Description of Additional Supplementary Files [file 41467_2019_13433_MOESM3_ESM.pdf]

Description of Additional Supplementary Files

File Name: Supplementary Data 1
Description: Single CpG associations.

File Name: Supplementary Data 2
Description: Significant differentially methylated loci, ordered by increasing P-value.

File Name: Supplementary Data 3
Description: Methylation-Gene Expression correlations.

File Name: Supplementary Data 4
Description: Kwashiorkor associated human phenotype ontologies.

File Name: Supplementary Data 5
Description: Study Gene Ontologies.

File Name: Supplementary Data 6
Description: MeQTL regions with significant slope interactions.

File Name: Supplementary Data 7
Description: Putative nutrition-sensitive meQTL SNPs that strongly alter likelihood of transcription factor binding.

File Name: Supplementary Data 8
Description: Non-nutrition-sensitive meQTL SNPs that strongly alter likelihood of transcription factor binding.

File Name: Supplementary Data 9
Description: Correlation between buccal and blood at differentially methylated loci.
