# Supplementary material for: Edematous severe acute malnutrition is characterized by hypomethylation of DNA
Source: Nat Commun. 2019 Dec 19;10:5791. doi: 10.1038/s41467-019-13433-6 (PMC6923441; doi:10.1038/s41467-019-13433-6)
Supplement: Supplementary file 13 — Reporting Summary [file 41467_2019_13433_MOESM13_ESM.pdf]

nature research

Corresponding author(s): Neil A. Hanchard

Last updated by author(s): Oct 11, 2019

Reporting Summary

Nature Research wishes to improve the reproducibility of the work that we publish. This form provides structure for consistency and transparency in reporting. For further information on Nature Research policies, see Authors & Referees and the Editorial Policy Checklist.

Statistics

For all statistical analyses, confirm that the following items are present in the figure legend, table legend, main text, or Methods section.

n/a	Confirmed	
☐	☒	The exact sample size (*n*) for each experimental group/condition, given as a discrete number and unit of measurement
☐	☒	A statement on whether measurements were taken from distinct samples or whether the same sample was measured repeatedly
☐	☒	The statistical test(s) used AND whether they are one- or two-sided<br>*Only common tests should be described solely by name; describe more complex techniques in the Methods section.*
☐	☒	A description of all covariates tested
☐	☒	A description of any assumptions or corrections, such as tests of normality and adjustment for multiple comparisons
☐	☒	A full description of the statistical parameters including central tendency (e.g. means) or other basic estimates (e.g. regression coefficient) AND variation (e.g. standard deviation) or associated estimates of uncertainty (e.g. confidence intervals)
☐	☒	For null hypothesis testing, the test statistic (e.g. *F*, *t*, *r*) with confidence intervals, effect sizes, degrees of freedom and *P* value noted<br>*Give P values as exact values whenever suitable.*
☒	☐	For Bayesian analysis, information on the choice of priors and Markov chain Monte Carlo settings
☒	☐	For hierarchical and complex designs, identification of the appropriate level for tests and full reporting of outcomes
☐	☒	Estimates of effect sizes (e.g. Cohen's *d*, Pearson's *r*), indicating how they were calculated

Our web collection on statistics for biologists contains articles on many of the points above.

Software and code

Policy information about availability of computer code

Data collection	None used
Data analysis	The R package ChAMP was used to process files with raw fluorescence intensity values from the methylation arrays and assess systematic biases; the Methylation Module in Illumina's GenomeStudio was used for array data normalization; further normalization and quality control was performed using the R package lumi; ConsensusPathDB was used for gene ontology and pathway enrichment analysis; genotype was called with Illumina's GenomeStudio software; PLINK was used to evaluate shared ancestry between genotyped individuals; the R package MatrixEQTL was used to identify meQTLs; the R package MotifBreakR was used to identify transcription factor binding motifs. Gene expression levels were background corrected and normalized on Affymetrix's Expression Console software (build 1.4.1.46) using the SST-RMA algorithm. All remaining statistical analyses, including linear regression, were perfomed using R's built in functions. Scripts used to analyze human phenotype and gene ontologies are available through the lab's gitHub repository.

For manuscripts utilizing custom algorithms or software that are central to the research but not yet described in published literature, software must be made available to editors/reviewers. We strongly encourage code deposition in a community repository (e.g. GitHub). See the Nature Research guidelines for submitting code & software for further information.

Data

Policy information about availability of data

All manuscripts must include a data availability statement. This statement should provide the following information, where applicable:

- Accession codes, unique identifiers, or web links for publicly available datasets
- A list of figures that have associated raw data
- A description of any restrictions on data availability

Methylation datasets generated during the current study are available on the Gene Expression Omnibus (GEO) database under the accession number GSE112893 [https://www.ncbi.nlm.nih.gov/geo/query/acc.cgi?acc=GSE112893]. The source data underlying Figure 3a and c, Supplementary Figure 5a-d, and Supplementary Table 1 are provided as a Source Data file. All relevant data are also available from the authors upon request.

Field-specific reporting

Please select the one below that is the best fit for your research. If you are not sure, read the appropriate sections before making your selection.

☒ Life sciences ☐ Behavioural & social sciences ☐ Ecological, evolutionary & environmental sciences

For a reference copy of the document with all sections, see nature.com/documents/nr-reporting-summary-flat.pdf

Life sciences study design

All studies must disclose on these points even when the disclosure is negative.

Sample size	Based on our preliminary data we anticipated that the most relevant CpGs would have at least a 5% difference in methylation between ESAM and NESAM. The standard deviation of methylation at each probe on the array varies with the degree of methylation, but a conservative estimate is 0.03. Taking a variably methylated site with a beta value of 60% and assuming a type I error rate of 0.5 x 10^-5 in each population, DC sample sizes of ~50 – 100 cases and controls in each population group have > 95% power to determine differential methylation at that locus. Ultimately, we were able to obtain 164 case and 145 control samples of adequate quality, resulting in an even greater power to detect methylation differences.
Data exclusions	Out of 407 samples analyzed on the DNA methylation microarray, 33 samples did not pass quality control (>2% failed probes) and were thus excluded. Of the 28 samples that were analyzed for correlation between DNA methylation and gene expression, eight did not pass default quality control filters and were excluded.
Replication	To verify reproducibility, we assessed and reported methylation results in each of the two geographically distant locations from which samples were recruited. We also demonstrate strong correlation between methylation assessed by microarray and that assessed by methylation sequencing at the same CpG sites .
Randomization	Severely malnourished children were categorized as cases or controls based on the presence or absence of nutritional edema, respectively. A random number generator was then used to assign samples to plates ahead of methylation array typing.
Blinding	There was no blinding during sample collection, since the process of sample allocation relied on medical examination and categorization based on the presence or absence of edema.

Reporting for specific materials, systems and methods

We require information from authors about some types of materials, experimental systems and methods used in many studies. Here, indicate whether each material, system or method listed is relevant to your study. If you are not sure if a list item applies to your research, read the appropriate section before selecting a response.

Materials & experimental systems

n/a	Involved in the study	
☒	☐	Antibodies
☒	☐	Eukaryotic cell lines
☒	☐	Palaeontology
☒	☐	Animals and other organisms
☐	☒	Human research participants
☒	☐	Clinical data

Methods

n/a	Involved in the study	
☒	☐	ChIP-seq
☒	☐	Flow cytometry
☒	☐	MRI-based neuroimaging

Human research participants

Policy information about studies involving human research participants

Population characteristics	Male and female participants with acute or a history of severe acute childhood malnutrition (SAM) were recruited from two countries. Individuals with acute malnutrition were between 0.25-3.58 years old, individuals with a history of SAM were between 17.08-49.58 years old. Participants were categorized as either cases and controls depending on the presence or absence of edema with concomitant severe malnutrition. Weight, age, and co-morbid illnesses were recorded at the time of recruitment. [see Supplementary Information]
Recruitment	Participants with severe acute childhood malnutrition (SAM) were recruited through the Tropical Metabolism Research Unit (TMRU) of the Caribbean Institute for Health Research (CAIHR) at the University Hospital of the West Indies (UHWI), located in St. Andrew, Jamaica. Adult participants with a history of severe malnutrition were recruited through TMRU/CAIHR. Participants with SAM were recruited from five Southern rural study sites in Malawi. [Detailed in "Experimental Design"]
Ethics oversight	National Health Science Review Committee (NHSRC) of the Ministry of Health, Government of Malawi; Ethics Committees of the UHWI/University of the West Indies Faculty of Medical Sciences, Kingston, Jamaica; Institutional Review Board of Baylor College

of Medicine, Houston, Texas, USA

Note that full information on the approval of the study protocol must also be provided in the manuscript.
